# VCFshiny: an R/Shiny application for interactively analyzing and visualizing genetic variants

**DOI:** 10.1093/bioadv/vbad107

**Published:** 2023-08-26

**Authors:** Tao Chen, Chengcheng Tang, Wei Zheng, Yanan Qian, Min Chen, Qingjian Zou, Yinge Jin, Kepin Wang, Xiaoqing Zhou, Shixue Gou, Liangxue Lai

**Affiliations:** Guangdong Provincial Key Laboratory of Large Animal Models for Biomedicine, South China Institute of Large Animal Models for Biomedicine, School of Biotechnology and Health Sciences, Wuyi University, Jiangmen 529020, China; Guangdong Provincial Key Laboratory of Large Animal Models for Biomedicine, South China Institute of Large Animal Models for Biomedicine, School of Biotechnology and Health Sciences, Wuyi University, Jiangmen 529020, China; Guangdong Provincial Key Laboratory of Large Animal Models for Biomedicine, South China Institute of Large Animal Models for Biomedicine, School of Biotechnology and Health Sciences, Wuyi University, Jiangmen 529020, China; CAS Key Laboratory of Regenerative Biology, Guangdong Provincial Key Laboratory of Stem Cell and Regenerative Medicine, Guangzhou Institutes of Biomedicine and Health, Chinese Academy of Sciences, Guangzhou 510530, China; Guangdong Provincial Key Laboratory of Large Animal Models for Biomedicine, South China Institute of Large Animal Models for Biomedicine, School of Biotechnology and Health Sciences, Wuyi University, Jiangmen 529020, China; Guangdong Provincial Key Laboratory of Large Animal Models for Biomedicine, South China Institute of Large Animal Models for Biomedicine, School of Biotechnology and Health Sciences, Wuyi University, Jiangmen 529020, China; Guangdong Provincial Key Laboratory of Large Animal Models for Biomedicine, South China Institute of Large Animal Models for Biomedicine, School of Biotechnology and Health Sciences, Wuyi University, Jiangmen 529020, China; CAS Key Laboratory of Regenerative Biology, Guangdong Provincial Key Laboratory of Stem Cell and Regenerative Medicine, Guangzhou Institutes of Biomedicine and Health, Chinese Academy of Sciences, Guangzhou 510530, China; Sanya Institute of Swine Resource, Hainan Provincial Research Centre of Laboratory Animals, Sanya 572000, China; Guangdong Provincial Key Laboratory of Large Animal Models for Biomedicine, South China Institute of Large Animal Models for Biomedicine, School of Biotechnology and Health Sciences, Wuyi University, Jiangmen 529020, China; CAS Key Laboratory of Regenerative Biology, Guangdong Provincial Key Laboratory of Stem Cell and Regenerative Medicine, Guangzhou Institutes of Biomedicine and Health, Chinese Academy of Sciences, Guangzhou 510530, China; Sanya Institute of Swine Resource, Hainan Provincial Research Centre of Laboratory Animals, Sanya 572000, China; Guangzhou National Laboratory, Guangzhou 510005, China; Guangdong Provincial Key Laboratory of Large Animal Models for Biomedicine, South China Institute of Large Animal Models for Biomedicine, School of Biotechnology and Health Sciences, Wuyi University, Jiangmen 529020, China; CAS Key Laboratory of Regenerative Biology, Guangdong Provincial Key Laboratory of Stem Cell and Regenerative Medicine, Guangzhou Institutes of Biomedicine and Health, Chinese Academy of Sciences, Guangzhou 510530, China; Sanya Institute of Swine Resource, Hainan Provincial Research Centre of Laboratory Animals, Sanya 572000, China

## Abstract

**Summary:**

Next-generation sequencing generates variants that are typically documented in variant call format (VCF) files. However, comprehensively examining variant information from VCF files can pose a significant challenge for researchers lacking bioinformatics and programming expertise. To address this issue, we introduce VCFshiny, an R package that features a user-friendly web interface enabling interactive annotation, interpretation, and visualization of variant information stored in VCF files. VCFshiny offers two annotation methods, Annovar and VariantAnnotation, to add annotations such as genes or functional impact. Annotated VCF files are deemed acceptable inputs for the purpose of summarizing and visualizing variant information. This includes the total number of variants, overlaps across sample replicates, base alterations of single nucleotides, length distributions of insertions and deletions (indels), high-frequency mutated genes, variant distribution in the genome and of genome features, variants in cancer driver genes, and cancer mutational signatures. VCFshiny serves to enhance the intelligibility of VCF files by offering an interactive web interface for analysis and visualization.

**Availability and implementation:**

The source code is available under an MIT open source license at https://github.com/123xiaochen/VCFshiny with documentation at https://123xiaochen.github.io/VCFshiny.

## 1 Introduction

Recent advances in sequencing technologies have enabled the detection of a large number of genetic variants at the whole genome level (Metzker 2010, van Dijk *et al.* 2014). Genetic variants are obtained in cells during acquired development, and these variants may be caused by DNA replication errors or exposure to environmental mutagens (Pei *et al.* 2021). The most common scenario for genetic variant detection is in cancer genomics research because most cancers are caused by genetic variants in driving genes, and harmful genetic variants continue to accumulate during the development of cancer (Nakagawa and Fujita 2018, Xiao *et al.* 2021). Thus, the crucial first step in the analysis of cancer sequencing data is identifying genetic variants (Koboldt 2020). Another use for genetic variant detection is in gene editing research because the wide application of clinical gene therapy has led to increasing concerns about its safety. The off-target effects of CRISPR/Cas9-mediated gene editing may bring potential risks (Kuscu *et al.* 2014, Aquino-Jarquin 2021, Höijer *et al.* 2022). Therefore, genetic variant detection could be used as an unbiased method of detecting off-target effects at the whole genome level(Veres *et al.* 2014, Kim *et al.* 2015, Wang *et al.* 2021, Luo *et al.* 2023). The 1000 Genomes Project and 100 000 Genomes project set out to provide a comprehensive description of common human genetic variation by applying whole-genome sequencing to a diverse set of individuals from multiple populations (Siva 2008, Peplow 2016). These studies described the distribution of genetic variation across the global sample, and discuss the implications for common disease studies. Re-analysis genetic variant data generated by these projects may also lead to new biological insights.

To identify mutations in DNA sequencing data, a series of variant callers and computational pipelines have been developed with their own unique characteristics (Barnell *et al.* 2019, Cameron *et al.* 2019, Krusche *et al.* 2019, Koboldt 2020, He *et al.* 2021). Despite differences in calling algorithms and applications, most use genome sequencing data aligned to a reference as input and output single nucleotide variants and indels recorded in variant call format (VCF) (Danecek *et al.* 2011). The VCF file stores the details of variations, including the chromosome location, base sequence, base quality, read depth, and genotype. An annotated VCF file, such as that annotated by Annovar (Wang *et al.* 2010), also has information columns containing the corresponding gene name and corresponding genomic features. The VCF files are usually used by end-users to search for variants of interest and evaluate the potential impact of these variants. Although some command line tools have been developed to filter, annotate, and visualize VCF files, these programs may require programming skills and a bioinformatics background, limiting their use by researchers without a computational background.

Recently, many efforts have been made to develop graphical tools to process VCF files for researchers with limited bioinformatics backgrounds. Tools such as vcfView (O'Sullivan and Seoighe 2020), VCF/Plotein (Ossio *et al.* 2019), shinyCircos (Yu *et al.* 2018), shinyChromosome (Yu *et al.* 2019), BrowseVCF (Salatino and Ramraj 2017), and IGV (Thorvaldsdottir *et al.* 2013) have been developed to enable researchers to browse and filter variants in the VCF. However, they skip the annotation step, so users may need to annotate the VCF file with other annotation tools prior to use. Other tools, including VCF-Server (Jiang *et al.* 2019), VCF-Miner (Hart *et al.* 2016), and Ensembl-VEP (McLaren *et al.* 2016), focus on annotating and filtering variants but lack visualization functions for exploring the variant information. And, some of these tools are obsolete and lack maintenance, making them unavailable. In addition, a major disadvantage of web tool solutions such as VEP is that the transmission of large amounts of genetic data over public networks raises confidentiality and performance issues and requires a dedicated server that may not be available to every end user.

To fill this void, we developed VCFshiny, an interactive R/Shiny application for analyzing and visualizing VCF files. It allows non-bioinformatician researchers to upload VCF files to annotate and visualize detailed variant information without requiring any programming code. VCFshiny allows users to annotate VCF files using Annovar or VariantAnnotation with commonly used databases. VCFshiny also accepts annotated VCF files for comparing and visualizing variants between different samples. Furthermore, VCFshiny supports the summarization of cancer driver gene-relevant variants and cancer mutational signatures, improving its ability to predict the biological consequences of variants. Collectively, it enables researchers without a bioinformatics background to explore and interpret variant data, thereby facilitating research in the field of genetics.

## 2 Features

**Figure 1.**
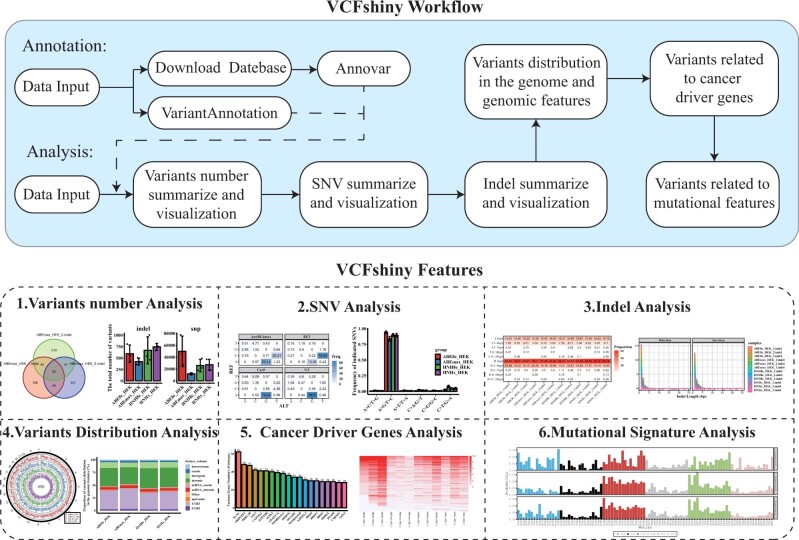
Overview of the full workflow performed by VCFshiny (annotation and visualization of genetic variant data analysis). (A) The analysis pipeline consists of two function modules: (i) variant annotation, and (ii) variant data analysis. Variant annotation module is supported by Annovar and VariantAnnotation, allowing users to download annotation database (such as dbsnp) and annotate variants to corresponding genes, genomic regions, or related disease. The variant data analysis module allows users to summarize the detailed information of VCFs and perform statistical analysis and comparison of variants between samples. (B) Result visualization. Once the analysis is done, user can interactively explore and export the results. For example, they can explore the total variant numbers (B1), base substitution bias of single nucleotides (B2), length distributions bias of indels (B3), location of variant in the genome and genome features (B4), variants in cancer driver genes (B5), and cancer mutational signatures (B6). The variant dataset used in this figure is an RNA-seq data of three breast cancer subtypes (TNBC, Non-TNBC, and HER2-positive) and normal human breast organoids (epithelium) samples (NBS) under the GEO accession number: GSE52194 (Horvath *et al.* 2013).

The VCFshiny workflow is illustrated in Fig. 1. This includes variant data annotation, summarization, and visualization.

### 2.1 Variant annotation

VCFshiny starts with uploading compressed VCF files, which should be filtered and annotated variation data but preferably not raw variant data with a large file size. For non-annotated VCF files, VCFshiny supplies Annovar (Wang *et al.* 2010), and VariantAnnotation (Obenchain *et al.* 2014) to annotate variants to genes or genomic regions. To easily query the commonly used database for variant annotation, VCFshiny also provides a web interface for users to download the database supported by Annovar. To use VariantAnnotation to annotate variants, users may need to manually install dependent database packages such as “TxDb.Hsapiens.UCSC.hg38.knownGene”. The annotated results are presented in a data table format for users to browse and download.

### 2.2 Variant summarization and visualization

VCFshiny summarizes the total number of genome-level variants of different samples and visualizes them by a bar chart. A Venn diagram is provided to show the overlap of variations between the biological replicates of the samples. To compare the variant deviation of single nucleotides, we calculate the base substitution frequencies against the total SNV background of each sample and visualize them via a heatmap and bar chart. To assess the distribution bias of indel length, we split insertion and deletion sizes into different ranges, calculate the frequencies of each sample under those ranges and visualize the results via a heatmap or histogram. Circos plots are one of the most efficient approaches to visualize the distribution of variations in the genome. VCFshiny uses the circlize package (Gu *et al.* 2014) to draw Circos plots. Furthermore, VCFshiny also summarizes the variation distribution frequencies among genomic feature regions, such as exonic, intronic, and intergenic regions.

### 2.3 Cancer-relevant variant analysis

To facilitate VCFshiny usage in cancer genetics, we provide functions to summarize cancer driver gene-relevant variants and cancer mutational signatures. We download 568 cancer driver genes from IntoGene (https://www.intogen.org/search), count the variant numbers located in these cancer driver genes, and visualize them via a bar plot and heatmap. Mutational signals are the result of a mutational process consisting of some form of DNA damage and subsequently acted upon by DNA repair or replication mechanisms. These mutational signals reveal both endogenous and exogenous factors in the development of cancer (Alexandrov *et al.* 2020). VCFshiny provides an analysis of mutant characteristics by the software package Musicatk (Chevalier *et al.* 2021).

### 2.4 Comparison and document

To highlight the advantages, we systematically compared VCFshiny with eight existing analysis and visualization software tools. VCFshiny fills some gaps in existing software and implements a number of enhanced features that make it an even more potentially freely available tool (Table 1). Detailed documentation and examples can be found in the package manual at https://123xiaochen.github.io/VCFshiny and Supplementary Materials.

**Table 1.** Comparison of applications for analyzing, filtering, annotating and visualizing VCF files.

	VCFshiny	vcfView	VCF-Server	VCF/Plotein	shinyCircos	shinyChromosome	BrowseVCF	VCF-Miner	IGV	Ensembl-VEP
**Does not require command line knowledge** Requires knowledge of command line commands by the user, either to install the application or to operate it.	✓	✓	✓	✓	✓	✓	✗	✗	✓	✗
**Graphical user interface** The program has a graphical user interface to make it easy for the user to interact, analyze and visualize information	✓	✓	✓	✓	✓	✓	✓	✓	✓	✓
**Custom VCF** The program allows to use an user provided VCF	✓	✓	✓	✓	✓	✓	✓	✓	✓	✓
**No pre-processing steps** The program does not require the VCF to be pre-processed or to be converted into a database format	✓	✓	✓	✓	✓	✓	✗	✗	✗	✓
**Data annotation** This program is able to variation data VCF file based on a variety of database comments	✓	✗	✓	✗	✗	✗	✗	✓	✗	✓
**Sample repeatability analysis** This program can detect the quality of sample duplication for multiple repeated experimental groups	✓	✗	✗	✗	✗	✗	✗	✗	✗	✗
**SNV analysis** This program can screen SNV data in mutation data and analyze the type and frequency of SNV mutations	✓	✗	✗	✗	✗	✗	✗	✗	✗	✗
**Indel analysis** This program can screen Indel data in the variation data and analyze the mutation length and frequency of Indel mutations	✓	✗	✗	✗	✗	✗	✗	✗	✗	✗
**Genomic circosplot** This program can show the distribution of SNVS and Indel on the genome map	✓	✗	✗	✗	✓	✗	✗	✗	✗	✗
**Genomic feature analysis** The program is able to analyze the functional characteristic area where the variation is located	✓	✗	✗	✗	✗	✗	✗	✗	✗	✓
**Key gene screening** The program can screen for high-frequency mutated genes in each VCF file based on the genomic feature	✓	✗	✓	✓	✗	✗	✓	✓	✗	✗
**Screening for cancer driver genes** The software can screen potential cancer drivers in conjunction with a cancer database	✓	✓	✗	✗	✗	✗	✗	✗	✗	✗
**Mutational signature analysis** This software can be used to select samples and analyze the mutation signature	✓	✓	✗	✗	✗	✗	✗	✗	✗	✗
**Available for free** The program can be used freely	✓	✓	✓	✓	✓	✓	✓	✓	✓	✓

## 3 Implementation

VCFshiny was developed based on the Shiny package on the R (R Development Core Team 2014) platform, which enables interactive web applications to be built directly from R code (Jia *et al.* 2022). VCFshiny integrates a number of different publicly available R packages to generate a convenient interface and computational efficiency to smoothly process variation data. The source code of VCFshiny was deployed on GitHub, and users can download it for free at https://github.com/123xiaochen/VCFshiny. VCFshiny can be launched and run directly with a single R function, “startVCFshiny()”, after installation.

## 4 Conclusions

We present an R/Shiny application, VCFshiny, that offers a user-friendly web interface to explore and visualize genetic variant information stored in VCF files. It allows users to annotate, filter, visualize, and export variants without any programming knowledge requirement. Owing to its user-friendly interface, VCFshiny can be used by both bioinformaticians and non-bioinformaticians experts. VCFshiny is under active development and maintenance and is available as an R package at https://github.com/123xiaochen/VCF-shiny.

## Supplementary Material

vbad107_Supplementary_Data

## Data Availability

The RNA-seq data used in this study is a public dataset under the GEO accession number: GSE52194, and the data source has been referenced in figure legend.
