# Supplementary material for: VCFshiny: an R/Shiny application for interactively analyzing and visualizing genetic variants
Source: Bioinform Adv. 2023 Aug 26;3(1):vbad107. doi: 10.1093/bioadv/vbad107 (PMC10493178; doi:10.1093/bioadv/vbad107)
Supplement: vbad107_Supplementary_Data [file vbad107_supplementary_data.zip › VCFshiny-user-manual.pdf]

A Tutorial for VCFshiny

1. Introduction

VCFshiny is an R shiny application that can be easily launched from a local web browser to analyze sequenced mutated data for use by scientists without programming expertise. In this tutorial, we will go through the installation and usage of each module step by step using the example dataset we provided at github page (https://github.com/123xiaochen/VCFshiny).

2. How to start

This is an instruction of how to install and run VCFshiny software locally (https://github.com/123xiaochen/VCFshiny).

Requirement:

- R (>= 4.2.0)
- Shiny (>= 1.6.0)

Getting Star:

- Open R and run the following command.

```
install.package("shiny")
devtools::install_github("123xiaochen/VCFshiny")
library(shiny)
library(VCFshiny)
VCFshiny::startVCFshiny()
```

(The first model of VCFshiny setting page will pop-up, see **Figure S1**).

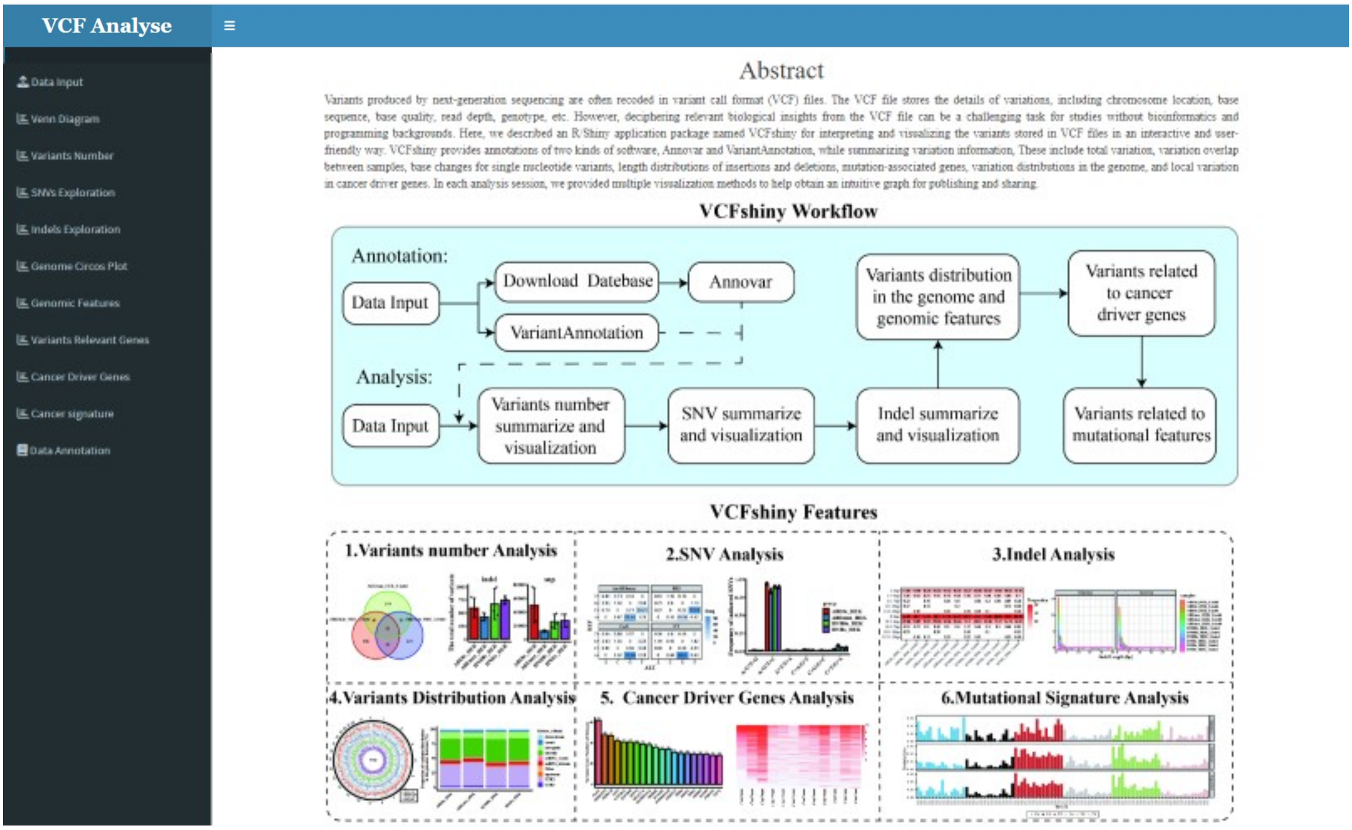

Figure S1: VCFshiny software GUI setting page

3. VCFshiny setting page

After starting the VCFshiny, there are a Introduction page and nine modules: (1) Data Input; (2) Variants Numbers; (3) SNP Analysis; (4) Indel Analysis; (5) Venn Diagram; (6) Circos Plot; (7) Genomic Feature; (8) Highest Variants Genes and (9) Cancer Driver Genes on the left panel of VCFshiny (**Figure S1**). For question and bug report, please leave your comment under issue section at github page (https://github.com/123xiaochen/VCFshiny).

4. Prepare data

In this section, we will introduce how to prepare two different input data sets:

1) Source of VCF input data

The Variant Call Format (VCF) is used to record gene sequence variations. It is also the first file format to be understood for genome population correlation analysis. First, the whole genome sequencing file is mapping to the reference, and then the resulting bam file is comprehensively analyzed using variant calling software such as GATK and the reference genome data to produce the VCF result.

2) Source of TXT input data

TXT files are one of several output formats annotated by Annovar (Wang K, Li M, Hakonarson H. 2010), which is able to analyze genetic variations in various genomes using the

latest data. Since the input data VCF file of Annovar software only contains the starting position of the mutation, it is necessary to adjust the input data before using, and add the end position of the mutation after the actual position of the mutation. Gene-based annotations reveal variant's direct relationship with known genes and its functional impact, while region-based annotations reveal Variant's relationship with specific segments of different genomes.

3) Input file name Requirements

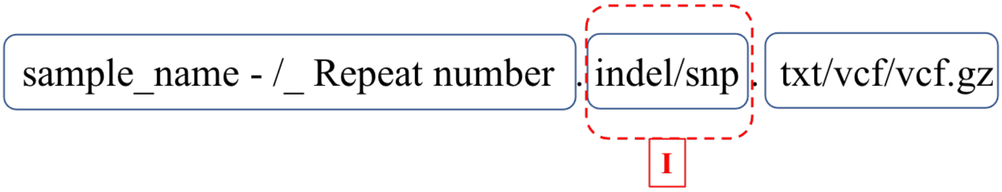

Figure S2: Input file name

- The first box represents the sample name, which can be the group of experiments and the number of repetitions, connected by the character "-" or "_".
- The second box represents the data type, which can be snp or indel data. When snp and indel are not classified in the data, this box can be absent (**Figure S2**-I).
- The third box represents the data format, which can be vcf files or vcf compressed files, and Annovar annotated TXT files.
- The contents of the three boxes are connected by ".".

4) Input compress files requirements

Before uploading the data to VCFshiny, that needs to be compressed. The following are the naming requirements for the compressed folder.

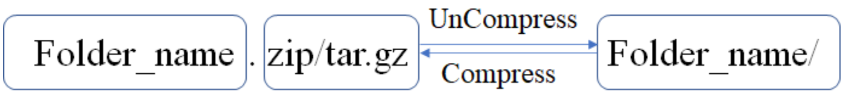

Figure S3: Input compress files name

- The compressed file name must be the same as the name of the compressed folder.
- The compressed file can be in *.tar.gz or *.zip format.

5) Example data set in VCFshiny software

We provide a built-in dataset that can be used to explore VCFshiny. The data set can be directly loaded into the APP by clicking the button "Use example data?" of the data input module (**Figure S4**-II). The built-in dataset was derived from the sequencing data of published articles, including a control group and three experimental groups, with three replicates in each group (Liang Y, Xie J, Zhang Q, et al. 2022). The sequencing data was first mapping to the reference, followed by GATK Variants calling and Annovar annotations.

5. Run VCFshiny

In this section, we will introduce step by step instruction in each module using the example built-in dataset provided at VCFshiny.

Module 1: Data Input

The first module contains (1) Choose the species of upload data (**Figure S4**-I); (2) Built-in dataset upload (**Figure S4**-II); (3) Upload user's dataset (**Figure S4**-III); (4) Upload data Button (**Figure S4**-IV); (5) Dataset select to display (**Figure S4**-V). User can choose to use their own dataset or built-in dataset to upload and select data to display.

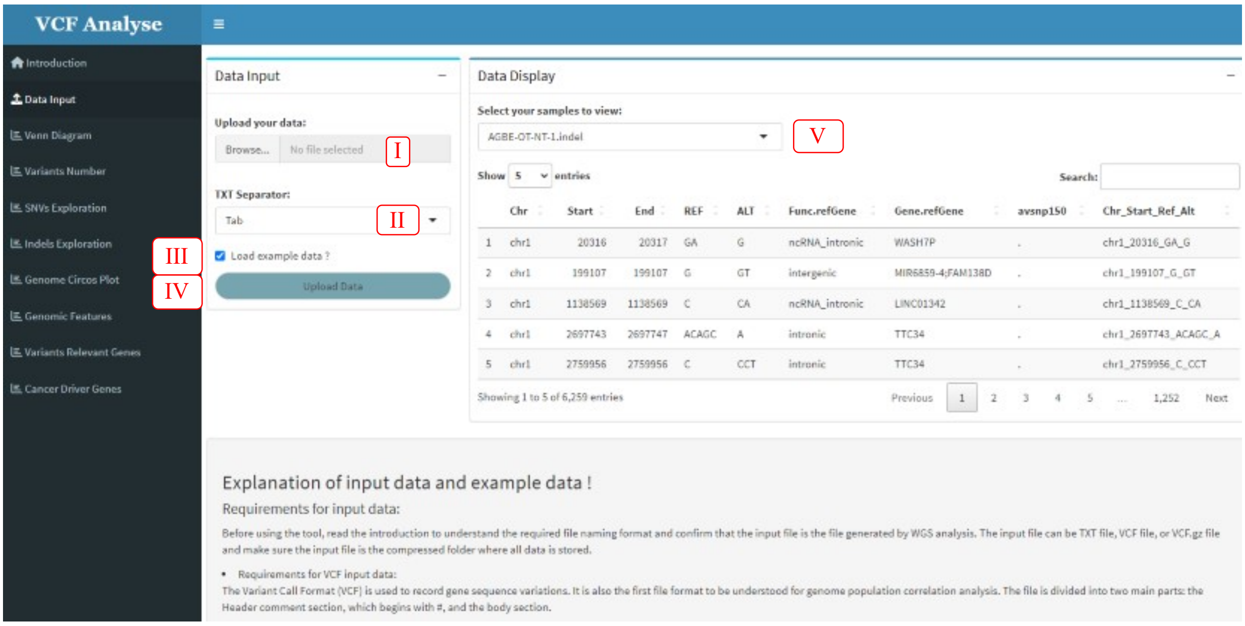

Figure S4: Data input page.

Module 2: Venn Diagram

In order to detect the duplicate data credibility of each group, users can cross-analyze the data using the Venn R package (Dusa, Adrian. 2021) and present the visualized results. This module consists of three parts: (1) The first part can select analysis data and setting visual parameter. Users can select SNP or Indel and interested groups for analysis, and parameters of Venn diagram can be set according to the provided button (**Figure S5**-I); (2) The second part is to visualize the cross data through Venn graph, and download the picture using the download button (**Figure S5**-II); (3) The third part "Display Venn Data" shows the cross data and can download the data through the download button (F**igure S5**-III).

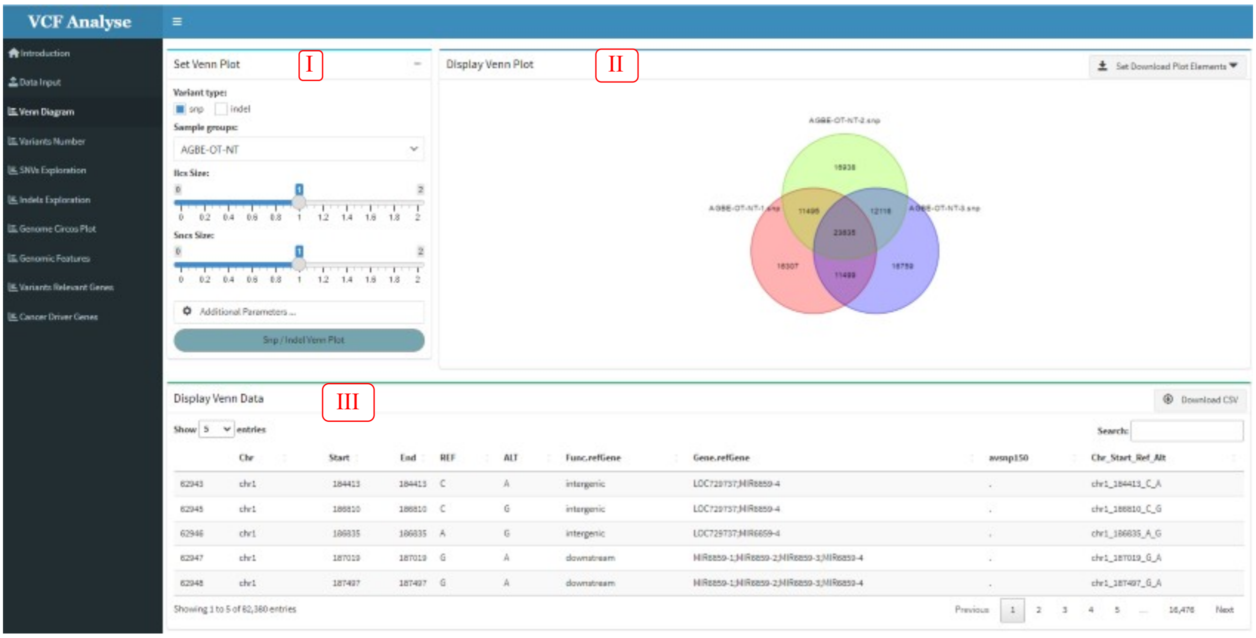

Figure S5: Venn Diagram page

<u>Module 3: Variants Number</u>

After uploading the data input, the uploaded input will be automatically divided into SNP and Indel according to the group. The main purpose of the analysis on the third module "Variants Number" is to display the number of SNP and Indel. This page contains three parts: (1) Set Summary Plot (**Figure S6**-I); (2) Display SNP + Indel ALL Variants Plot (**Figure S6**-II); (3) Display SNP + Indel ALL Variants Data (**Figure S6**-III).

In the "Set Summary Plot" part, user can adjust the display chart according to the parameters, including whether to display the value label, the font size in the chart and the width of the bar plot (**Figure S6**-I). In addition, clicking the "Additional Parameters …" button will pop up some other setting parameters, including "Error bar width", "Error bar size", "Jitter size" and "Jitter width", At the same time, users with programming ability can adjust the plot by adding ggplot2 drawing command (**Figure S6**-I). after the parameters are set and run by the user, the ggplot2 R package is used to draw the bar plot and Display in the section of "Display SNP + Indel ALL variables plot". Download button is added to this part. After clicking the download button, setting download parameters will pop up, including "Download Plot Width" and "Download Plot Height" and Download button (**Figure S6**-II). in addition to the picture display, the module also includes the data display part "Display SNP + Indel ALL Variants Data", and provides the output of the data (**Figure S6**-III).

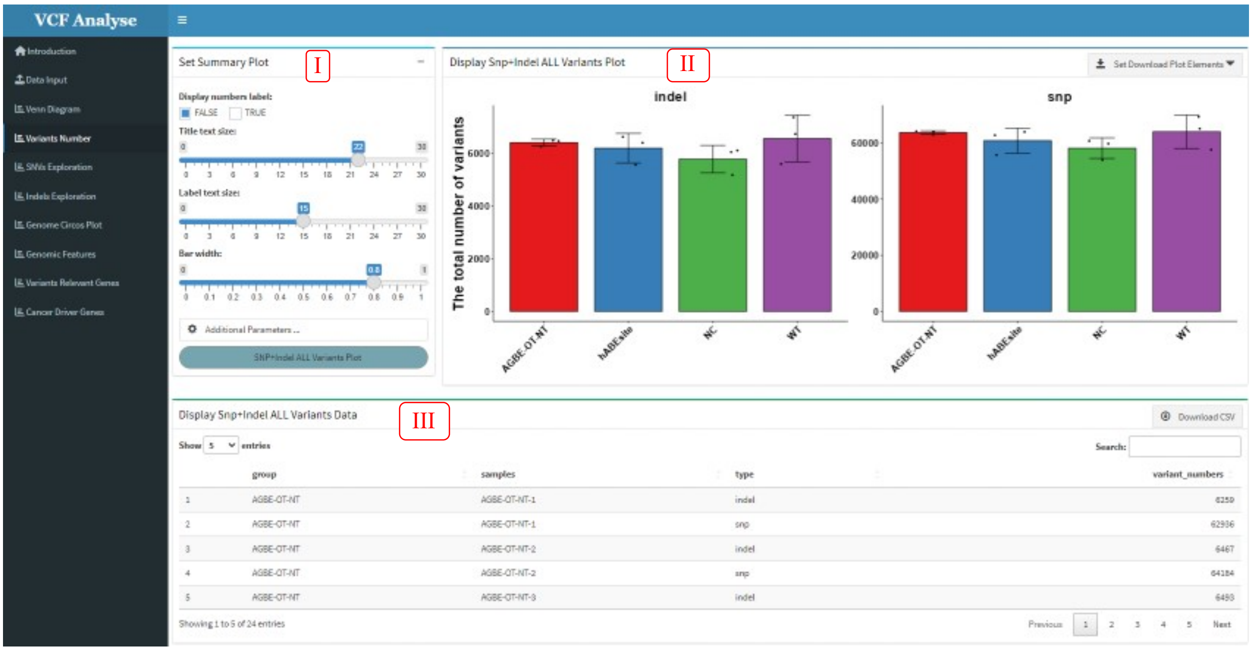

Figure S6: Variants number page

Module 4: SNP Analysis

This module is to analyze and use heatmap and bar plot to display SNP data of different groups, through which different mutation rates of each group can be clearly seen. This module includes three parts: (1) In the first part "Set SNP Analysis Plot", users can choose drawing methods, including heat map and bar chart (**Figure S7** and **Figure S8**). The bar chart includes two stacking methods (**Figure S9**), and users can choose interested experimental group and plotting parameters. (**Figure S7**-I and **Figure S8**-I); (2) The second part "Display SNP Analysis Plot" can display the SNP data use heatmap or bar plot, and user can use download button to download plot (**Figure S7**-II and **Figure S8**-II); (3) The third part "Display SNP Analysis Data" can display the heatmap or bar plot data, and also provides the output of the data (**Figure S7**-III and **Figure S8**-III).

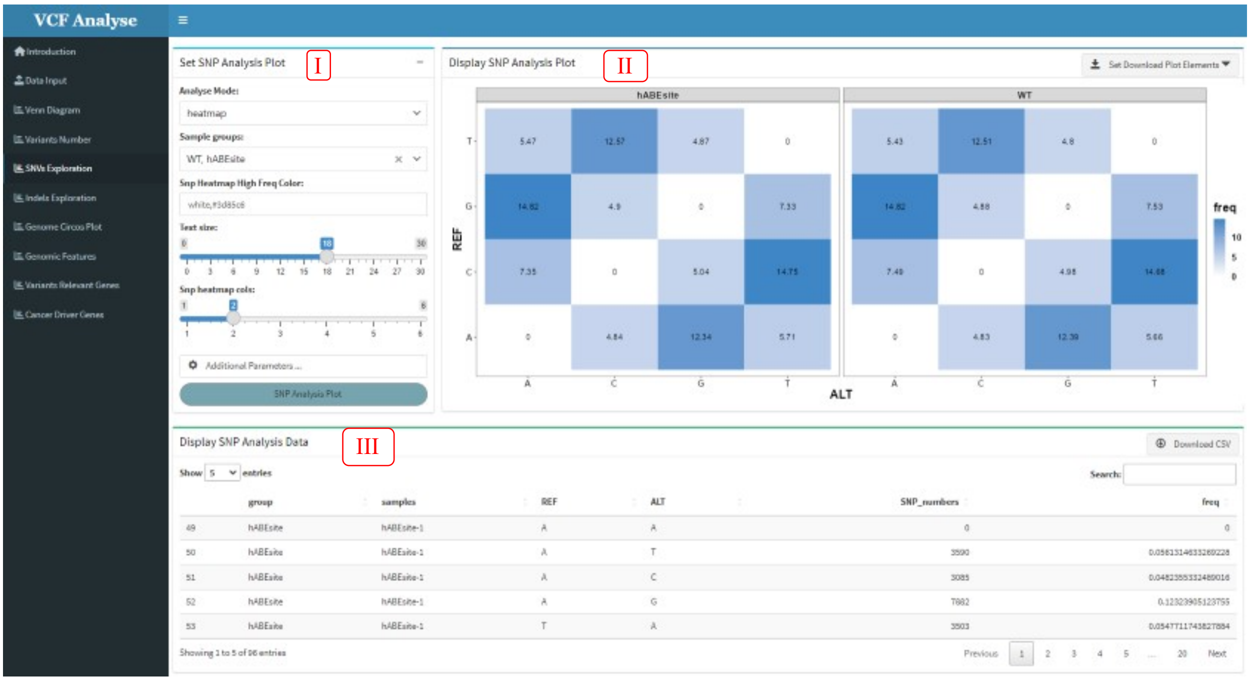

Figure S7: SNP Analysis (Heatmap) page

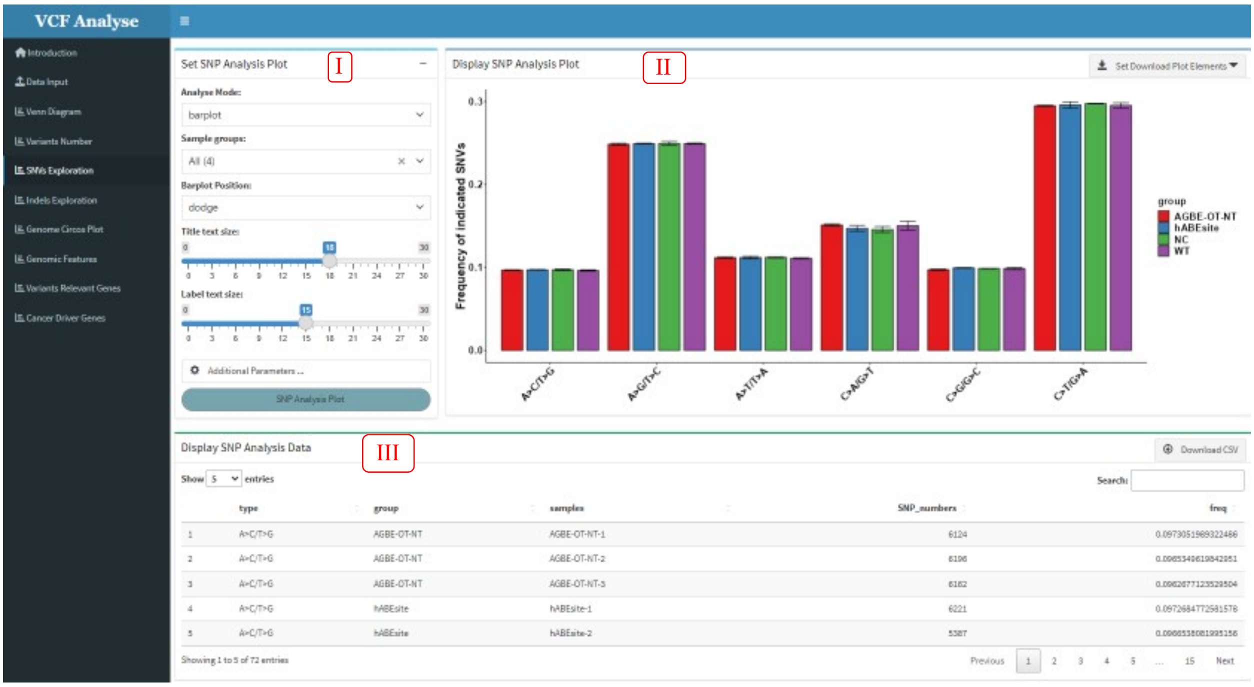

Figure S8: SNP Analysis (Barplot) page

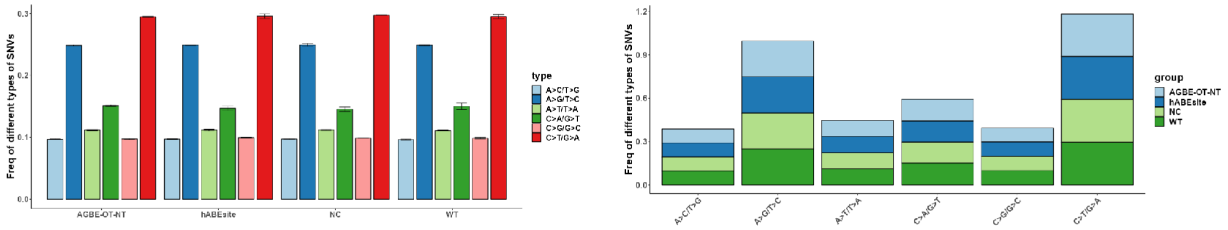

Barplot Position = "dodge"

Barplot Position = "fill"

Figure S9: Two types of bar plot

Model 5: Indel Analysis

This module uses two mapping methods of Indel length density distribution map and Indel length heat map to display Indel data. Through this method, we can intuitively see the situation of various types of mutations in different populations. This module consists of three parts: (1) In the first part “Set Indel Analysis Plot”, users can choose drawing methods, including density plot and heatmap plot (**Figure S10** and **Figure S11**), in which users can choose interested experimental groups and drawing parameters (**Figure S10**-I and **Figure S11**-I); (2) The second part “Display Indel Analysis Plot” can display the Indel data use density plot or heatmap, and user can use download button to download plot (**Figure S10**-II and **Figure S11**-II); (3) The third part “Display Indel Analysis Data” can display the density plot or heatmap data, and also provides the output of the data (**Figure S10**-III and **Figure S11**-III).

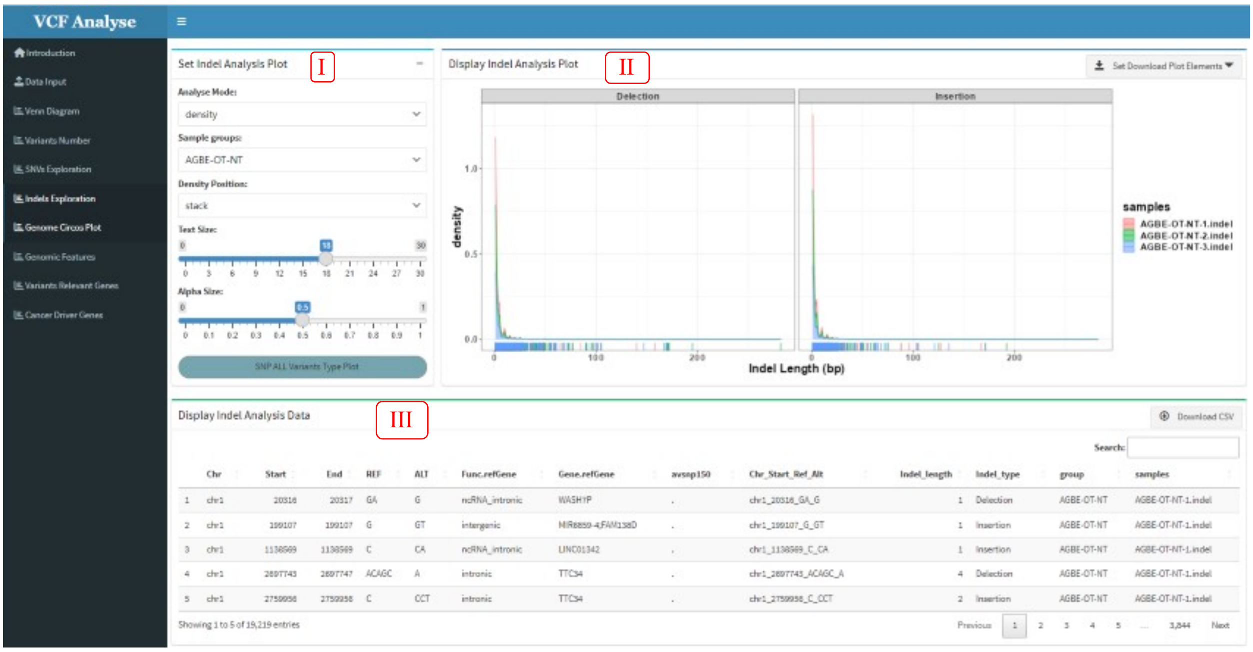

Figure S10: Indel Analysis (density plot) page

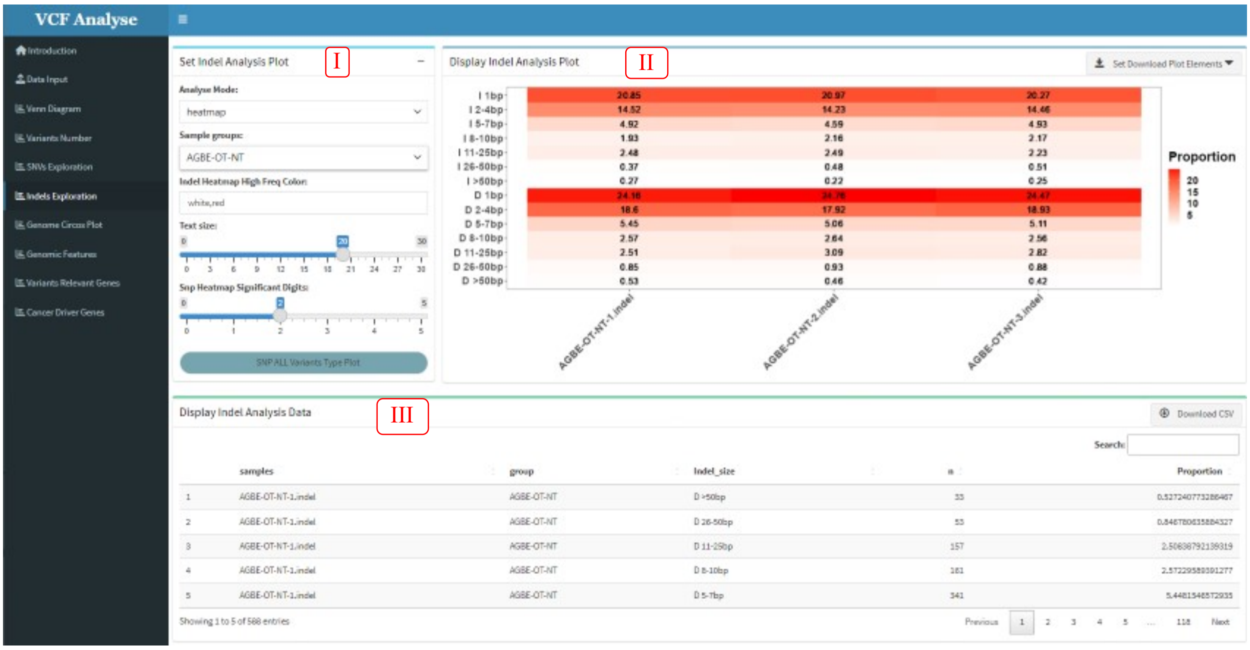

Figure S11: Indel Analysis (heatmap) page

Module 6: Circos Plot

In order to observe the distribution of SNP or Indel on chromosomes, the "Circos Plot" module uses the circlize R package (Gu, Z.et al. 2014) to plot the selected data on a chromosomal circle plot according to the location of mutations. This module consists of three parts: (1) The first part is "Set Circle Plot", it can select analysis data and setting drawing parameters. Users can select SNP or Indel data for analysis. This module sets three display elements, namely point, line and rectangle (**Figure S13A**-I), and can set the position of labels in Circos chart according to the label position button provided. In addition, there are some additional parameters for users to set (**Figure S13A**-II); (2) The second part is "Set Circle Elements Plot", which can set the display elements, including the color and size of the elements represented by each group (**Figure S13B**); (3) The third part is "Display Circle Plot", which mainly visualizes the data according to the parameter Settings in the previous two steps. Users can use the download button to download the visual results (**Figure S13A**-III).

A:

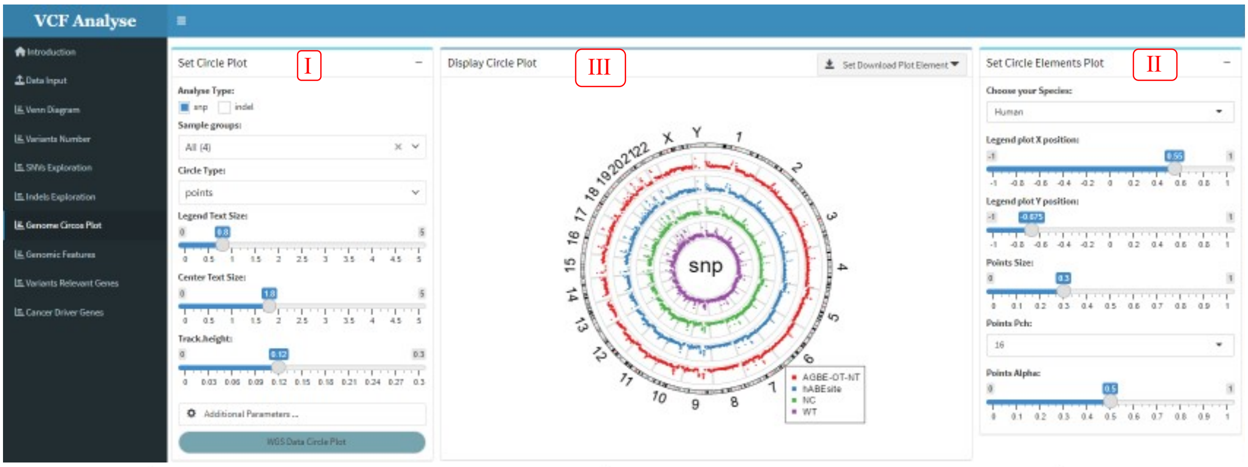

B:

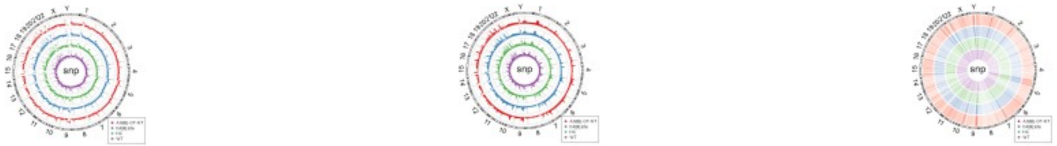

Circle Type: Points Circle Type: Lines Circle Type: heatmap

Figure S13: Circos Plot Page

Module 7: Genomic Feature (annotated data)

In order to detect the specific position of SNP or Indel in the genome, the “Genomic Feature” module makes a statistical analysis of the annotated file according to the genome where the annotated mutation is located, and displays the results with a bar chart. This module includes three parts: (1) The first part is “Set Distribution Plot”, it can select analysis data and setting drawing parameters. Users can select SNP or Indel data and interested groups for analysis. The bar chart provides "fill" and "dodge" stacking modes (**Figure S15**), for users to choose by themselves. Meanwhile, users need to select the column of genomic function position in the data according to different data. In addition, additional parameter Settings are also set in this part, which users can set according to their own needs (**Figure S14**-I); (2) The second part “Display Distribution Plot” can display the distribution data use bar plot, and user can use download button to download plot (**Figure S14**-II); (3) The third part “Display Distribution Data” can display the bar plot data, and also provides the output of the data (**Figure S14**-III).

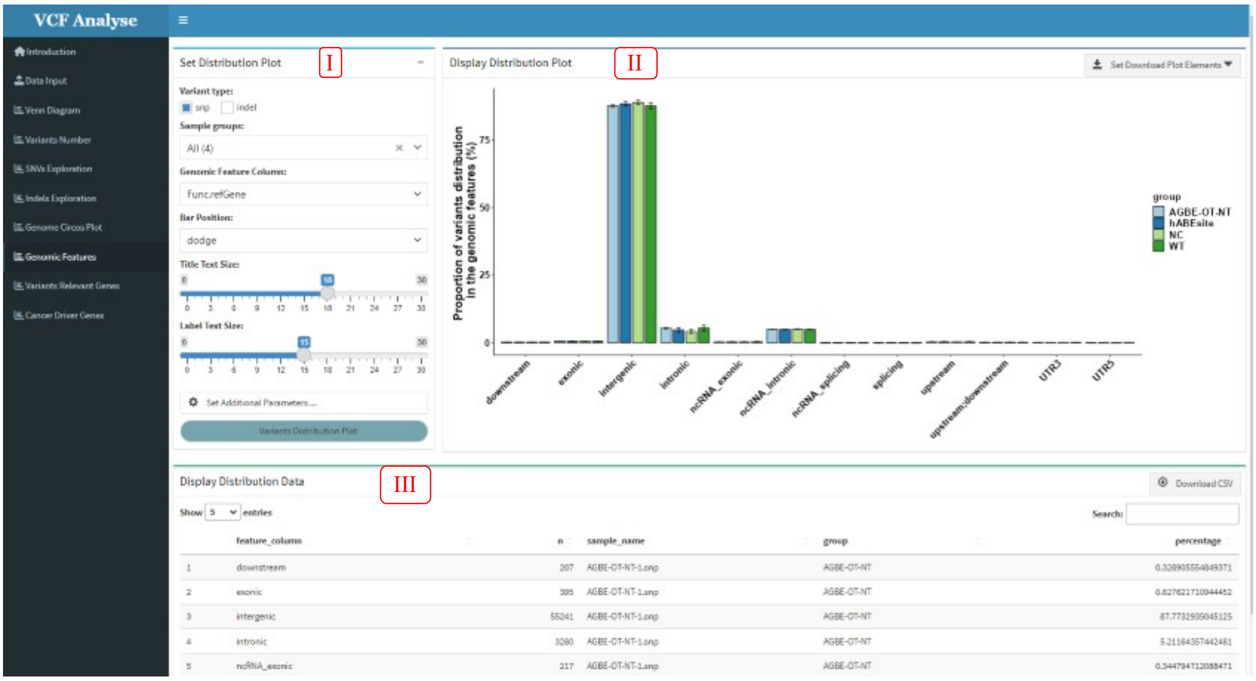

Figure S14: Genomic Feature page

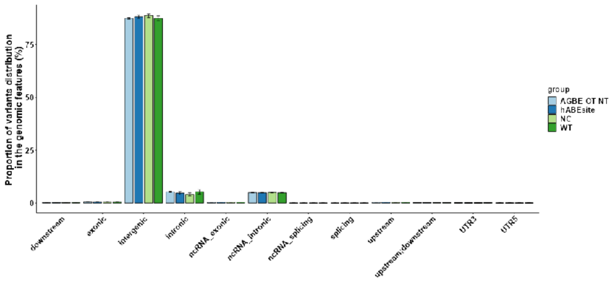

position = "dodge"

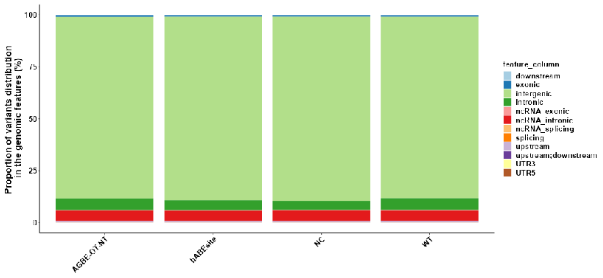

position = "fill"

Figure S15: Two bar position of Genomic Feature

Module 8: Highest Variant Genes

In order to visually see the situation of highest mutant genes in each sample, this "Highest Variant Genes" module makes statistics on the highest mutant genes in each sample and displays the statistical results. This module includes three parts: (1) The first part “Set Variants Genes Plot” is mainly about the selection of data and the setting of drawing parameters. Users can select the type of data, the position of mutation on the genome and the sample according to their own needs, and then carry out the next analysis. At the same time, users need to select the column of genomic functional location in the data and the gene functional location to be analyzed according to different data. In addition, additional parameter Settings are also set in this part, which users can set according to their own needs (**Figure S16**-I); (2) The second part “Display Variants Genes Plot” can display the Highest mutation data use bar plot, and user can use download button to download plot (**Figure S16**-II); (3) The third part “Display Variants Genes Data” can display the bar plot data, and also provides the output of the data (**Figure S16**-III).

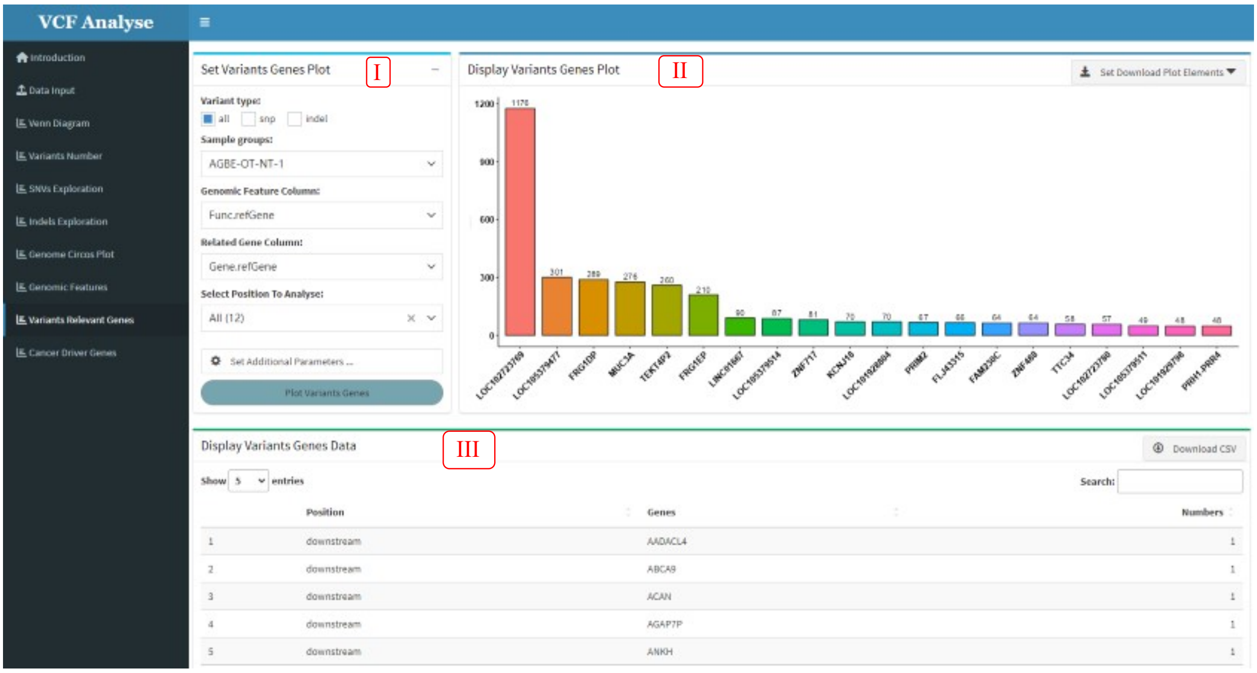

Figure S16: Highest Variant Genes page

<u>Module 9: Cancer Driver Genes</u>

After the statistics of highly mutated genes in all samples are carried out in Module 8, users can then use this module to select interested samples for heat map comparative analysis and screen out cancer driver genes. This module includes three parts: (1) The first part is the selection of data and the setting of drawing parameters. Users can select data types and analysis samples according to their needs, and then select the column of gene function in the data and the gene function region to be analyzed according to their needs. At the same time, this part also sets additional parameters, which users can set by themselves (**Figure S17**-I); (2) The second part can display the different samples Highest mutation data use heatmap, and user can use download button to download plot (**Figure S17**-II); (3) The third part can display the heatmap data, and also provides the output of the data (**Figure S17**-III).

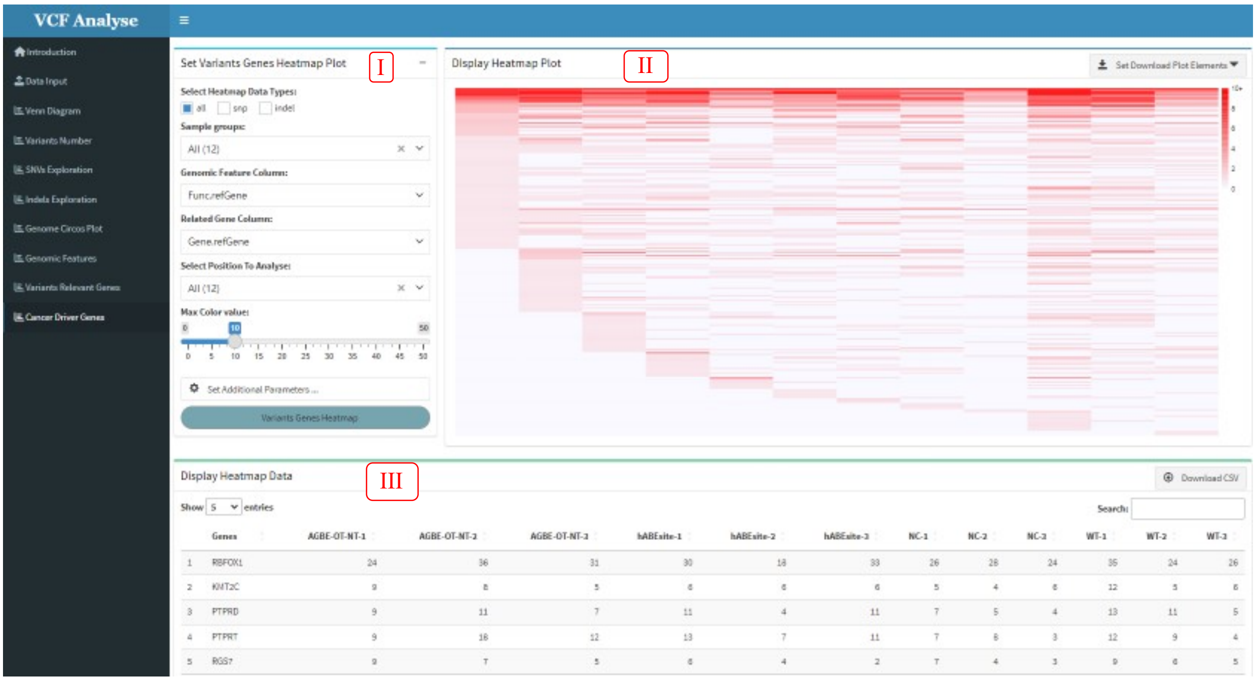

Figure S17: Cancer Driver Genes page

Module 10: Annotation Part

VCFshiny not only provides analysis of mutated data after annotation, but also provides user friendly annotation features. VCFshiny provides two kinds of software, *Annovar* and *VariantAnnotation*, to annotate human variant VCF files. After downloading the annotation results, they can be uploaded directly to the analysis part for analysis.

➢ **Annovar Annotation**

ANNOVAR is an efficient software tool for functional annotation of genetic variation detected in the genome using up-to-date information. Given a list of chromosomal variants, starting positions, ending positions, reference nucleotides, and observed nucleotides, ANNOVAR can perform annotation in three ways: 1. Gene-based annotation: determines whether SNPs or CNVs cause protein coding changes and the affected amino acids. 2. Region-based annotation: Identifying variations in specific genomic regions. 3. Filter-based annotation: Identify variations of records in a particular database.

The first Annotation tool provided by VCFshiny for mutate Data is Annovar. Users can annotate mutant data through "Annovar Annotation" module in the "Data Annotation" page (**Figure S18**-I), which includes two parts: (1) The first part is data uploading and annotation parameter setting. Users can upload their own variation data and select annotation type and annotation database according to requirements (**Figure S18**-II). (2) The second part is the display part of annotation results. After data upload and annotation, annotation results will be displayed in the form of a table in the second part. At the same time, the built-in download button enables users to download annotation results for further analysis (**Figure S18**-III).

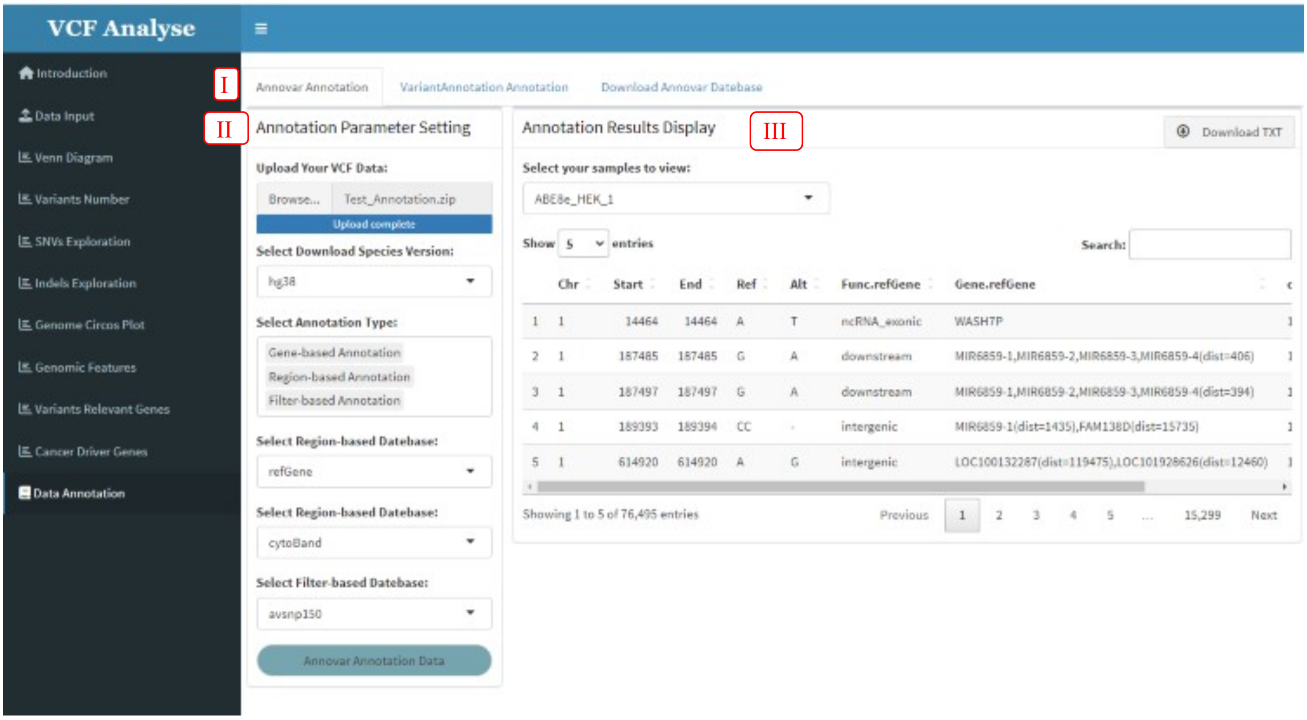

Figure S18: Annovar Annotation

- **VariantAnnotation Annotation**

VariantAnnotation is an *R / Bioconductor* package for the exploration and annotation of genetic variants. Capabilities exist for reading, writing and filtering variant call format (VCF) files.

The second Annotation tool provided by VCFshiny for variable Data is VariantAnnotation, which can be annotated by users in the “VariantAnnotation Annotation” module on the “Data Annotation” page (**Figure S19**-I), which includes two parts: (1) The first part is the reading of data and the selection of annotation parameters. Users can upload their own variation data and select annotation parameters for annotation (**Figure S19**-II). (2) The second part is the display part of annotation results. After data upload and annotation, annotation results will be displayed in the form of a table in the second part. At the same time, the built-in download button enables users to download annotation results for further analysis (**Figure S19**-III).

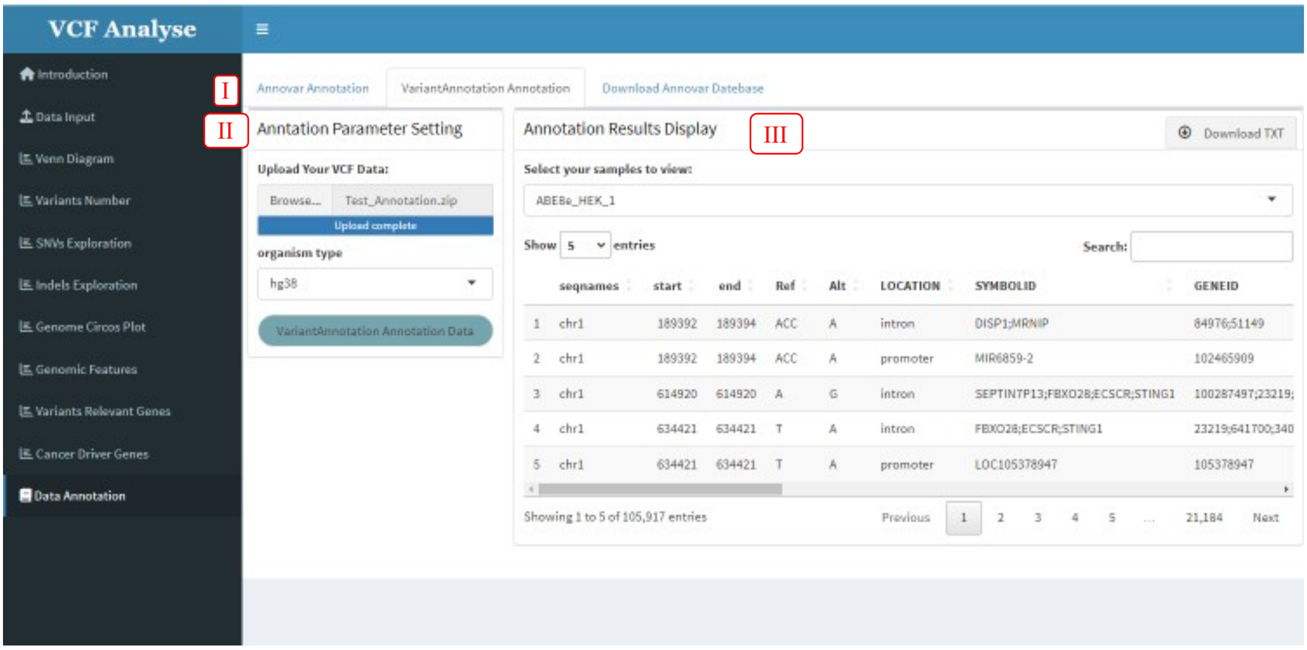

Figure S19: VariantAnnotaion Annotation

➢ **Download Annovar Database**

Before using Annovar annotation in VCFshiny, required annotation Database needs to be downloaded. The “Download Annovar Database” module in the “Data Annotation” page of VCFshiny provides a convenient download page (**Figure S20**-I). Users can download the database in Annovar software according to their own needs. This page includes three parts: (1) Download parameter selection section. Users can select the type and name of the database to download according to their needs (**Figure S20**-II). (2) Download the display part of the database, after the completion of the database download, the current downloaded database is displayed (**Figure S20**-III). (3) The database name provided by Annovar. We will display the downloaded data provided by Annovar, and users can download the database according to the display information (**Figure S20**-IV).

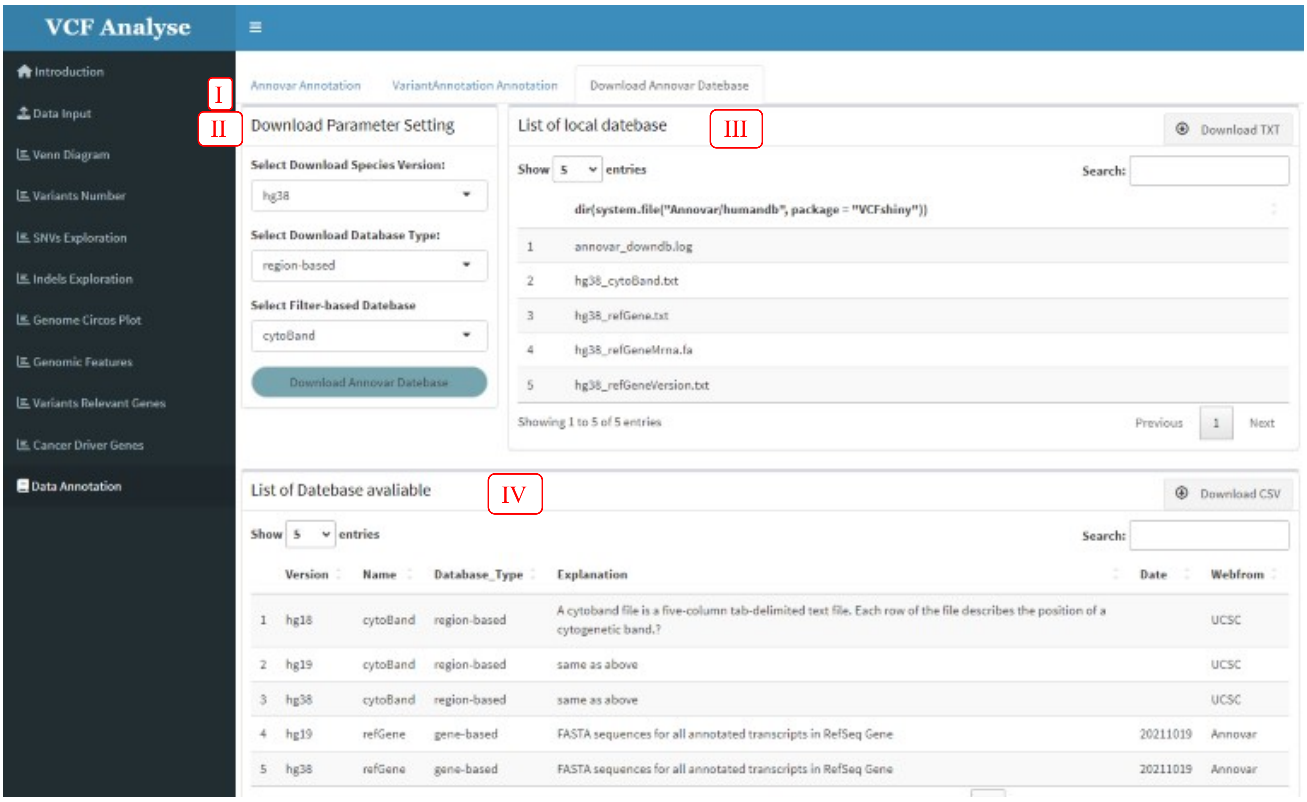

Figure S20: Download Annovar Database

Reference

Liang Y, Xie J, Zhang Q, et al. AGBE: a dual deaminase-mediated base editor by fusing CGBE with ABE for creating a saturated mutant population with multiple editing patterns. *Nucleic Acids Res*. 2022;50(9):5384-5399.

Wang K, Li M, Hakonarson H. ANNOVAR: functional annotation of genetic variants from high-throughput sequencing data. *Nucleic Acids Res*. 2010;38(16):e164.

Dusa, Adrian (2021): venn: Draw Venn Diagrams. Comprehensive R Archive Network. https://CRAN.R-project.org/package=venn

Gu, Z.et al. (2014) circlize implements and enhances circular visualization in R. Bioinformatics, 30, 2811–2812.
